# Complete chloroplast genome of the tree fern *Alsophila podophylla* (Cyatheaceae)

**DOI:** 10.1080/23802359.2017.1419095

**Published:** 2017-12-22

**Authors:** Shanshan Liu, Jingyao Ping, Zhen Wang, Ting Wang, Yingjuan Su

**Affiliations:** a School of Life Sciences, Sun Yat-sen University, Guangzhou, China;; b College of Life Sciences, South China Agricultural University, Guangzhou, China;; c College of Life Sciences, Nanjing Agricultural University, Nanjing, China;; d Research Institute of Sun Yat-sen University in Shenzhen, Shenzhen, China

**Keywords:** *Alsophila podophylla*, chloroplast genome, phylogenetic analysis

## Abstract

The chloroplast genome of the tree fern *Alsophila podophylla* has been completely sequenced. The genome is 166,151 bp in size and features a typical quadripartite structure with the large (LSC, 86,762 bp) and small single copy (SSC, 21,641 bp) regions separated by a pair of inverted repeats (IRs, 28,874 bp each). It encodes 133 genes including 91 protein-coding genes, 33 tRNA genes, eight rRNA genes, and one pseudogene. Maximum-likelihood tree indicates that *A. podophylla* is sister to *A. spinulosa*. This work provides a solid molecular resource for surveying phylogeny and chloroplast genomics of ferns.

*Alsophila podophylla* Hooker is a tree fern belonging to subgenus *Gymnosphaera* (genus *Alsophila*, Cyatheaceae), with a trunk height of 1–3 m (Zhang and Nishida 2013). It can distinguish from other tree ferns by its nearly entire pinnules, basal pinna with a long stalk, and lamina with a conform terminal pinna. As a relic species, the distribution range of *A. podophylla* had drastically reduced during the Quaternary glaciation events (Zhou et al. 2004; Su et al. 2005). In China, its extant individuals are mainly restricted to rain forests at an altitude of 350–700 m (Su et al. 2005; Zhang and Nishida 2013). *Alsophila podophylla* represents a core member to deal with the classification and phylogeny of Cyatheaceae (Ching 1978; Wang et al. 2003), acquirement of its whole chloroplast (cp) genome sequence will facilitate the chloroplast phylogenomics of Cyatheaceae.

Fresh leaves of *A. podophylla* were sampled from the living collection at South China Botanical Garden, Chinese Academy of Sciences (CAS). The specimen is stored in Herbarium of Sun Yat-sen University (SYS; voucher: *SS Liu 201610*). Genomic DNA was extracted using the Tiangen Plant Genomic DNA Kit (Tiangen Biotech Co., Beijing, China). Sequencing took place on the Hiseq 2500 platform (Illumina Inc., San Diego, CA), which generated a total of 7,222,454 raw reads. After quality assessment, clean reads were *de novo* assembled into contigs by Velvet (Zerbino and Birney 2008), which were further aligned and oriented with the cp genome of *Woodwardia unigemmata* (NC_028543) as a reference. All gaps were filled by PCR amplification. Annotations were performed using DOGMA (Wyman et al. 2004) and tRNAscan-SE programs (Lowe and Eddy 1997). The program MAFFT v7.311 (Katoh and Standley 2013) was used to create a multiple sequence alignment of the complete cp genome of *A. podophylla* with those of other nine plants downloaded from GenBank. A phylogenetic tree based on maximum-likelihood (ML) analysis was inferred using RAxML v.8.0 with 1000 bootstrap replicates (Stamatakis 2014).

The complete cp genome of *A. podophylla* is a circular DNA molecule of 166,151 bp in length (GenBank accession number: MG262389), which is the largest plastome among the studied ferns so far. It has a typical quadripartite structure with the large (LSC, 86,762 bp) and small single copy (SSC, 21,641 bp) regions separated by two identical inverted repeats (IRs, 28,874 bp each). The cpDNA contains 133 genes, including 91 protein-coding genes, eight ribosomal RNA genes, 33 tRNA genes, and one pseudogene. Among these genes, 118 are unique genes, and 14 are totally duplicated in IRs. The *ndhB* gene in IRb was identified as a pseudogene due to a fragment duplication in exon 2. In addition, 14 genes contain one intron, whereas three genes (*ycf3*, *clpP*, and *rps12*) have two introns. ML tree strongly supports that *A. podophylla* is the sister group to *A. spinulosa* (Figure 1); and they further form a monophyletic clade with the heterosporous fern *Marsilea crenata*. The cp genome of *A. podophylla* provides a reliable molecular resource for phylogenetic studies and chloroplast genomics of ferns.

**Figure 1.**
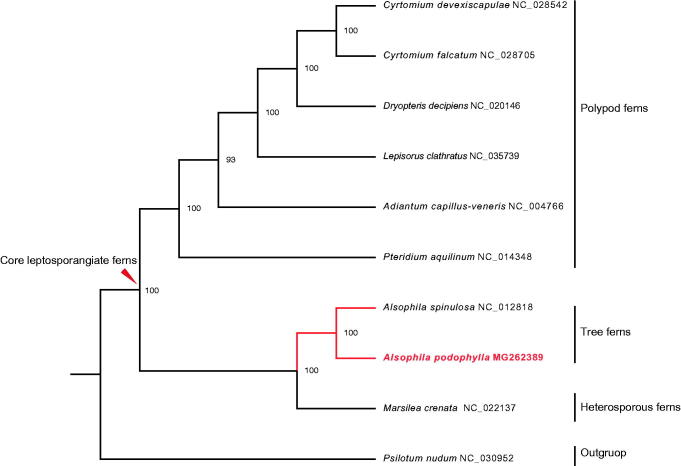
The maximum-likelihood phylogenetic tree based on the complete chloroplast genome sequences of nine monilophytes. *Psilotum nudum* is used as an outgroup. Numbers above the nodes are bootstrap values for 1000 replicates.
